# Knowledge and practice towards intravenous fluid therapy in children among nurses in the pediatrics emergency department of selected public hospitals

**DOI:** 10.1038/s41598-024-52921-8

**Published:** 2024-01-30

**Authors:** Garoma Gemechu Tolera, Birhanu Melaku Kasaye, Temesgen Beyene Abicho

**Affiliations:** 1 Department of Emergency and Critical Care Nursing, College of Health Science, Wallaga University, Nekemte, Ethiopia; 2 Department of Emergency Medicine, College of Health Science, Addis Ababa University, Addis Ababa, Ethiopia

**Keywords:** Paediatric research, Respiratory distress syndrome

## Abstract

Morbidity and mortality in hospitalized patients can be increased due to errors that are caused by inadequate knowledge and unsatisfactory practice of intravenous (IV) fluid therapy among healthcare workers. The knowledge and practice of nurses are very critical to IV fluid therapy because they are the cornerstone of a subject. This study assessed nurse's knowledge and practice of IV fluid therapy. A cross-sectional study design was employed at four selected public hospitals in Addis Ababa, Ethiopia. Data were collected from 112 nurses using a structured questionnaire for knowledge and using an observational checklist for practice. Data were analyzed using SPSS version 26 computer programs. Most respondents (67%) were males; the mean age of respondents was 31.2 ± 4.3. Among participated nurses, 42% (95% CI 32.8, 51.2) and 56.3% (95% CI 47.1, 65.6) had inadequate knowledge and satisfactory practice regarding IV fluid therapy in children, respectively. A significant association was observed between nurses' intravenous fluid therapy knowledge and in-service training that nurses who had training on fluid therapy in children had 4 times adequate knowledge than those who had no training (P = 0.01), an educational qualification that master degree holders had 4.8 times adequate knowledge than first-degree holders (P = 0.04) and training institution that nurse who had taken training in governmental teaching institution had 4 times adequate knowledge than who had taken training in private teaching institution (P = 0.011). No statistically significant association was found between practice level and independent variables regarding IV fluid therapy. Nurses’ knowledge of IV fluid therapy was inadequate and practice was relatively satisfactory. Continuous education and training of nurses on IV fluid therapy should be conducted regularly to improve their knowledge and practice. Further research should be employed involving other hospitals and focusing on risk factors for knowledge and practice inadequacy that are not discussed in this study.

## Introduction

Intravenous fluid therapy is among the most common routine nursing care procedures and has been practiced for more than 180 years globally^1^. It involves the administration of intravenous (IV) fluids, to nearly all hospitalized patients, for body fluid and electrolyte maintenance and as diluents for medications^2^. Relative benefits of crystalloids and colloid fluid resuscitation were controversial more than in the treatment of patients with hypovolemic shock^3^. Rapid intravenous fluid administration guidelines implementation contributed to the reduction of mortality due to hypovolemic shock over the years^4^.

Rapid and appropriate fluid resuscitation with crystalloids is very important to improve outcomes and to reduce the death of children in emergency departments as well as wards for admitted children. Different guidelines and management protocols recommend initial fluid bolus at a rate of 20 ml/kg of normal saline over 20 min^5,6^. Intravenous fluid therapy is a collaboration activity of health professionals, especially nurses and physicians. Nurses’ knowledge of age-specific considerations during the administration of IV fluids is critical in promoting patient safety and preventing complications for positive patient outcomes^7^.

About 70% of all intravenous administrations including fluid and medication had at least one clinical error that could be minimized by nurses' correct procedures and enough nurses. These errors have been associated with inadequate knowledge of nurses and other healthcare workers about IV fluid therapy^8^. According to a report by the National Institute of Health Care, Excellence guidance, 39% of intravenous fluid therapy errors were made by nurses only and 36% jointly by nurses and emergency physicians^9^.

According to empirical evidence globally, about 20% of the patients had errors in IV fluid therapy that are attributable to inadequate knowledge among healthcare workers including nurses (Enquiry, 2011^10^). Studies to assess the knowledge of nurses regarding intravenous therapy predominantly recorded that the majority of nurses had adequate knowledge of intravenous fluid therapy^11^. Poor knowledge of IV infusion decreases the quality for patients which can cause a poor outcome. According to one study conducted in Kenya, 17% of deaths among hospitalized patients were caused by inappropriate IV fluid therapy^12^.

The Royal College of Nursing (2016) standards of practice direct that the nurses involved in the administration of IV infusions should be trained in infusion therapy, including IV fluid types, clinical judgment assessment, administration, monitoring, and complications^13^. One descriptive study showed that there was no consensus regarding best practices to achieve pediatric fluid resuscitation goals among nurses, and other specialty and sub-specialty groups, and the study recommended further investigation of this problem^14^. The majority of the studies conducted globally, in Africa and Ethiopia did not address the title of this study rather they studied health professionals' knowledge and practice of fluid therapy in adults.

In Ethiopia, elsewhere, there is no study conducted on the assessment of nurses' knowledge and practice of intravenous fluid therapy in children. The purpose of this study was to describe nurses' knowledge and practice working in the pediatric emergency department regarding intravenous fluid therapy in children and to correlate between knowledge and practice level of nurses with selected independent variables. The study further aimed to establish the relationship between nurses' knowledge, practice, and sociodemographic characteristics. The study will provide important information for other researchers to conduct further studies focusing on risk factors for inadequacy of knowledge and unsatisfactory practice such as work overload, job satisfaction and promotion. Furthermore, based on the findings of the study the health facilities will take gap-filling actions.

## Materials and methods

### Study area

The study was conducted in selected public hospitals in Addis Ababa, capital city of Ethiopia. In Addis Ababa, there are 43 hospitals (11 public hospitals and 32 private hospitals) out of which selected for this study.

### Study design and study period

An institutional based cross-sectional study was conducted from March, 2022 to April, 2022.

### Population and eligibility criteria

The source population was all nurses working in the pediatric emergency department of public hospitals in Addis Ababa, Ethiopia, with a study population of all nurses working in the pediatric emergency department of selected hospitals. All nurses who had 6 months and more work experience, who were available during data collection, and who had the interest to participate were included in the study. However, nurses on annual and sick leave during the study period were excluded.

### Sampling procedure

There are 11 public hospital out of which four hospitals were selected purposively for this study by considering the pediatric emergency department they have and several nurses working in the department to get an adequate sample size. The consecutive sampling was used to collect data from all the nurses. Because of the small number of nurses working in the pediatric emergency department of selected hospitals, consecutive sampling is used to include all nurses and to get a relatively adequate sample size. The total number of nurses working in a pediatric emergency at selected hospitals was 126, 117 nurses participated in the study and 112 nurses responded to all questionnaires with a response rate of 95.7%.

Tikur-Anbessa Specialized Hospital = 42

St. Peters Hospital = 21

Zewuditu Memorial Hospital = 24

Yekatit 12 Hospital = 25

### Data collection procedure

The study tools were adopted from other literature^13,15^ with some modifications according to the objectives of the study to assess the knowledge and practice of nurses towards intravenous fluid therapy in children. The tools for data collection have three parts which were socio-demographic characteristics of study participants (9 items), knowledge (11 items), and practice (10 items observational checklists) of nurses regarding intravenous fluid therapy in children. There were 11 knowledge question items and the level of knowledge of nurses was calculated out of 29 because five questions had multiple responses. Tools were applied by using well-structured questionnaires through a self-administered response method for the knowledge test and by observation of nurses using observational checklists to assess practice. To assess the practice the nurses gave their consent but they were not allowed to know the contents of the observational checklist before being observed to minimize bias.

### Data quality control

The questionnaire was pre-tested by the principal investigator on 10% (n = 11) of the study population 2 weeks before actual data collection at St. Paulos Millennium Medical College Hospital to check its clarity, understandability, and reliability. Accordingly, the Cronbach's Alpha result of the pre-test was 0.710. Two days of short training were given for the data collectors and supervisors to enhance the quality of data and to ensure that all the data collectors have the same information about the study tools, and follow the same survey procedures. In addition, during the actual data collection process, supervisors were cross-checking consistency, completeness, and any missing data. Data cleaning for inconsistencies, missing values, and amendments were considered as needed before data analysis.

### Data management and analysis

Data were collected and checked for completeness and accuracy, then coded and entered by the investigator into the Epi data version 4.6 computer program. Subsequently, the data was transmitted to SPSS version 26 computer programs for analysis. Data analysis was performed using descriptive and inferential statistics. Univariate statistics were calculated to summarize respondents' socio-demographic characteristics knowledge and practice scores. Bivariate analysis was used to assess for statistically significant associate factors for knowledge and practice. Multivariate analysis was conducted for those variables that had a P value < 0.25 in binary logistic regression. A P value less than 0.05 was considered significant. The analyzed data were presented on charts, tables, and narrative text.

### Ethical approval

Ethical clearance was obtained from the Departmental of Emergency Medicine Research Ethics Committee of Addis Ababa University, College of Health Science, and submitted to hospitals. All methods were performed in accordance with the relevant guidelines and regulations of ethical procedures according to the Declaration of Helsinki with confidentiality and data privacy maintained to the maximum. After getting permission from these hospitals, informed consent was obtained after an information sheet on the objective of the study, the voluntariness of the participant, benefits, and risks of the study, and confidentiality of the study participant's information was provided to the participant nurses, and the collection of data was started.

### Operational definitions

#### Knowledge

Intravenous fluid therapy knowledge was measured as a two-score in the validated test for knowledge^16^.

***Adequate knowledge***: Nurses who score more than the mean knowledge score.

***Inadequate knowledge***: Nurses who score less than the mean knowledge score.

#### Practice

Intravenous fluid therapy practice was measured as a two-score in the validated test for practice^16^.

***Satisfactory practice***: Nurses who score more than the mean of practice score.

***Unsatisfactory practice:*** Nurses who score less than the mean of practice score.

## Result

### Sample characteristics of the respondents

Of the total 117 study participants, 112 were involved in this study with a response rate of 95.7%. Most respondents (67%) were males; the mean age of respondents was 31.2 with an SD of 4.3. About half (51.8%) had 6 months to 5 years of experience. The majority of them (72.3%) were first-degree holders, and only 32.1% of nurses had taken training regarding intravenous fluid therapy in children (Table 1).

**Table 1:** Socio-demographic characteristics of nurses working in the pediatric emergency department, Addis Ababa, 2022.

Variables	Categories	Frequency (N = 112)	Percent (%)
Age	20–29	42	37.5
	30–39	64	57.1
	40–49	6	5.4
Sex	Male	75	67.0
	Female	37	33.0
Marital status	Married	59	52.7
	Single	53	42.3
Qualification	Degree	81	72.3
	Masters	31	27.7
Institute	Government	76	67.9
	Private	36	32.1
Work experience (years)	1–5	58	51.8
	6–10	47	42.0
	11–15	6	5.2
	> 15	1	1
Training	Yes	36	32.1
	No	76	67.9
Protocol	Yes	75	67.0
	No	37	33.0

### Knowledge level of nurses

**Figure 1:**
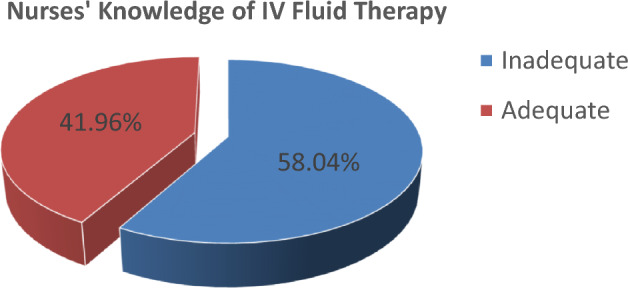
Distribution of samples according to the knowledge level regarding IV fluid therapy in children among nurses working in the emergency department, Addis Ababa, 2022.

Of all respondents 42% (95% CI 32.8%, 51.2%) of them had adequate knowledge whereas more than half (58%) of them had inadequate knowledge about intravenous fluid therapy in children by scoring more than and less than the mean score of knowledge, respectively (Fig. 1).

The minimum score for knowledge assessment was 8 (27.6%) and the maximum score was 24 (82.8%) out of 29 questions and by 100%, respectively. Over half of the nurses (58.9%, n = 66) correctly identified crystalloids as initial fluid therapy compared to colloids. Only 23.3 (n = 26) of them correctly selected Lactated Ringer's as the most physiological IV fluid than other types of IV fluid. Among all participants, only 21.4% (n = 24) were able to correctly identify at least three ions contained in Lactated Ringer's Solution. Only 15.2% (n = 17) of participants correctly identified the composition of maintenance fluid in IV fluid therapy. Regarding parameters to be monitored during IV fluid therapy, 22.3% (n = 25) of nurses correctly identified all listed options. Concerning the two body systems mainly monitored during IV fluid therapy 33.9% (n = 38) of them correctly selected circulatory and renal systems. The least correctly answered knowledge question was a complication of IV fluid therapy which was 9.8% (n = 11) (Table 2).

**Table 2:** Knowledge score result of nurses working in the pediatric emergency department of the selected public hospital, in Addis Ababa, Ethiopia, 2022 G.C.

Correct Answers	Frequency (%)
Crystalloids are used as initial fluid therapy in children	66 (58.9%)
Ringer lactate is the most physiological IV fluid	26 (23.2%)
Isotonic is defined as the fluid that has the same concentration of ions as sodium	70 (62.5%)
Potassium, sodium, and calcium ions that are found in Lactated Ringer’s solution	24 (21.4%)
24 h as a requirement for maintenance fluid for children	94 (83.9%)
Calculation of maintenance fluid in children is based on weight	105 (93.8%)
Components of maintenance fluid in children as normal saline, potassium, glucose, sodium	17 (15.2%)
Parameters to be monitored when administering IV fluids as infusion rate, cannula site infiltration, input–output of fluid, daily body weight	25 (22.3%)
Signs of fluid volume overload: increase in work of breathing, jugular vein distension, increase in pulse, hypoxemia, third spacing of fluid	14 (12.5%)
The two systems in the human body are mainly monitored to assess fluid balance: the circulatory and renal systems	38 (33.9%)
Complications of IV fluid therapy in children include: embolism, thrombophlebitis, infiltration and extravasation, fluid overload	11(9.8%)

### Practice level of nurses

**Figure 2:**
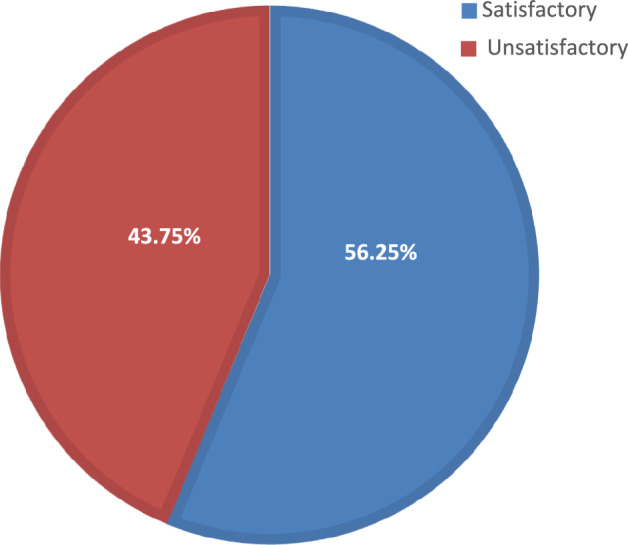
Distribution of samples according to the practice level regarding IV fluid therapy in children among nurses working in the emergency department, Addis Ababa, Ethiopia, 2022.

The practice level of nurses on IV fluid therapy in children was classified based on the mean score of practice as satisfactory (more than the mean) and unsatisfactory (less than the mean). Accordingly, more than half of the study respondents 56.3% (95% CI 47.1%, 65.6%) had satisfactory practice and the remaining 43.8% (n = 49) of them had an unsatisfactory practice of IV fluid therapy in children with 3 and 10 minimum and maximum scores, respectively (Fig. 2).

### Nurses' practice of IV fluid therapy in children

There were 10 observational checklists prepared to assess the level of nurses’ practice on intravenous fluid therapy in children. According to the result of practice observation, more than 85% of nurses checked the type and amount of IV fluid against the physician's order (89.3%) and accurately adjusted the flow rate (86.6%). Of all observed nurses, 69.6% and 63.4% checked for signs of infiltration and site for signs of phlebitis, respectively. Only 33.9% of observed nurses were inspecting the rate of flow and function of the IV line at least every hour. The least frequently practiced observation was checking respiration and auscultates lung sounds periodically which was seen in 25.9% of nurses (Table 3).

**Table 3:** Observed practice score of pediatric emergency department nurses on IV fluid therapy in children in selected public hospitals, in Addis Ababa, Ethiopia, 2022 G.C.

Checklists	Met (practiced)	
	Frequency (n)	Percent (%)
Check the type and amount of IV fluid against the physician's order	100	89.3
Document any additives which are added to the fluid	95	84.8
Identify the patient using at least two separate identifiers	79	70.5
Accurately adjust the flow rate	97	86.6
Inspect the rate of flow and function of the IV line at least every hour	38	33.9
Document the prescribed fluid on the chart	92	82.1
Look infusion site for signs of infiltration	78	69.6
Check the above IV insertion site for signs of phlebitis	71	63.4
Check pulse and blood pressure periodically	60	53.6
Check respiration, auscultate lung sounds periodically	29	25.9

### Factors associated with knowledge

Multivariate logistic regression analysis showed that nurses who had training on fluid therapy were 4.1 times more likely to belong to the adequate knowledge group than the inadequate knowledge group compared to those who had not taken training [AOR = 4.137; 95% CI (3993–0.241); P = 0.01]. Female respondents had 2.5 times more adequate knowledge than male respondents but the difference was not statistically significant at a P value < 0.05 [AOR = 2.092; 95% CI (0.940–4.656); P = 0.058]. The result further showed masters master-holder respondents had 4.8 times more adequate knowledge of IV fluid therapy in children compared with those who were first-degree owners which was statistically significant at P value < 0.05 [AOR = 4.838; 95 CI (1.654–14.153); P = 0.04]. Nurses who had graduated from the governmental institute had 4 times more adequate knowledge group than the inadequate knowledge group compared to those who had graduated from private institute [AOR = 4.042; 95% CI (1.382–11.822); P = 0.011] (Table 4).

**Table 4:** Associated factors analysis of knowledge among pediatric emergency department nurses, Addis Ababa, Ethiopia, 2022.

Variables group	Practice level		COR (CI, 95%)	P value	AOR (CI, 95%)	P value
	Satisfactory	Unsatisfactory				
Gender						0.805
Male	43	37	0.876 (0.397–1.933	0.742	0.899 (0.385–2.098)	
Female	20	17				
Age (years)						
≤ 30	33	19	1.737 (0.814–3.707)	**0.153**	0.453 (0.182–1.127)	0.088
> 30	30	30				
Marital status						
Single	30	23	1.028 (0.486–2.171)	0.943	0.999 (0.446–2.236)	0.998
Married	33	26				
Education level						
Degree	47	34	0.772 (0.336–1.772)	0.541	0.618 (0.239–1.594)	0.319
Masters	16	15				
Experience (years)						
≤ 5	34	24	0.819 (0.388–1.730)	0.600	1.189 (0.510–2.771)	0.689
> 5	29	25				
Institute						
Government	46	30	1.714 (0.770–3.813)	**0.187**	1.902 (0.782–4.622)	0.156
Private	17	19				
Training						
Yes	23	13	0.628 (0.278–1.420)	0.264	1.968 (0.757–5.113)	0.165
No	40	36				
Protocol						
Yes	45	30	0.632 (0.286–1.396)	0.256	0.809 (0.344–1.902)	0.627
No	18	19				

*Significant at P value < 0.05. Significant values are in bold.

### Factors associated with practice

The Association of practice level and independent variables was not statically significant at a P value < 0.05. However, some variables were more predictors than the other variables within the same group. Respondents who had work experience of ≤ 5 years had 1.2 times more satisfactory practice than those who had greater than 5 years of work experience [AOR = 1.189; 95% CI (0.239–1.594); P = 0.319], nurses who had taken training had 2 times more satisfactory skill of IV fluid therapy than those who had not taken training [AOR = 1.968; 95% CI (0.757–5.113); P = 0.165] and nurses who had been trained in the governmental teaching institute had about 2 times more satisfactory practice than those trained in private teaching facility [AOR = 1.902; 95% CI (0.782–4.622); P = 0.156] (Table 5).

**Table 5:** Associated factors analysis of practice among nurses working in the pediatric emergency department, Addis Ababa, Ethiopia, 2022. Significant values are in bold.

Variables group	Knowledge level (n = 112)			COR (CI, 95%)	P value	AOR (CI, 95%)	P value
	Adequate	Inadequate					
Sex							
Male	27		48	2.092 (0.940–4.656)	0.071		
Female	20		17				
Age ( years)							–
≤ 30	23		29	1.90 (0.561–2.525)	0.651		
> 30	24		36				
Marital status							–
Single	22		31	0.965 (0.455–2.047)	0.926		
Married	25		34				
Education level							
Degree	26		55	4.442 (1.832–10.772)	0.001*	4.838 (1.654–14.153)	0.004*
Masters	21		10				
Experience (years)							
≤ 5	28		30	1.123 (0.804–3.675)	0.162	0.581 (0.230–1.470)	0.252
> 5	19		35				
Institute							
Government	40		36	4.603 (1.798–11.789)	0.001*	4.042 (1.382–11.822)	0.011*
Private	7		29				
Training							
Yes	20		16	5.269 (1.011–15.089)	0.007*	4.137 (0.3993-0.241)	0.010*
No	27		49				
Protocol							
Yes	34		41	1.531 (6.78–3.451)	0.305	0.530 (0.187–1.501)	–
No	13		24				

## Discussion

The current study showed that only 41.96% of respondents had adequate knowledge of IV fluid therapy. In line with this, the study done in Nepal to assess nurses’ knowledge and practice regarding intravenous fluid therapy in children showed that only 49.1% of respondents had adequate knowledge of IV fluid therapy^16^. Contrary to this one study conducted in Dhaka city to assess the level of knowledge and practice on intravenous fluid therapy among staff nurses found that 54.7% had good knowledge of IV fluid therapy^17^. This difference could be attributed to that the researcher included only nurses having more than 2 years of work experience.

The finding of our study showed that the majority of nurses (58.04%) had inadequate knowledge of intravenous fluid therapy in children. This study result agrees with a study conducted in Iraq in which 63.3% of nurses had inadequate knowledge of intravenous fluid therapy^18^.

The current study showed that only 58.9% of respondents correctly selected crystalloid fluid for initial fluid therapy in children compared to colloids. Contrary to this, a study conducted in the Australia and Netherlands to assess fluid resuscitation in pediatrics showed that 98% of health professional respondents' first choice of fluid for pediatric resuscitation was crystalloid specifically normal saline^19^. The present study showed that only 21.4% of nurses correctly identified the ions composition of Lactated Ringer's solution. In line with this, a study performed in Kenya showed that very few (15.4%) of the participants were able to correctly identify a minimum of three ions^13^.

In the current study, there was no statistically significant association between years of experience and knowledge scores. Contrary to our finding one study conducted in Denmark to assess the knowledge of nurses about IV fluid therapy found that there was a significant association between years of experience of nurses and knowledge score^20^. This difference may have been due to more number of nurses specializing in emergency nursing after becoming registered nurses in the later study area.

In the present study, there was no statistically significant association between age group, sex, and knowledge scores. This result was supported by the study conducted in Kenya to assess nurses’ knowledge of IV therapy in which there was a negative correlation between respondents’ gender, age group, and IV fluid therapy knowledge at P < 0.05^13^.

The current study showed that educational qualification was a statistically significant predictor of the knowledge level of respondents at a P value < 0.05. The result of our finding contradicts the study conducted in Iraq to compare nurses’ knowledge and practice in which there was no significant association between educational qualification and the knowledge level of participants with a P < 0.05^21^. This disagreement may have been due to the presence of respondents holding master's levels in our study. Our study found that the knowledge score of nurses was significantly associated with in-service training on IV fluid therapy at a P value < 0.05. Likewise, a study conducted in Nepal to assess nurses' knowledge and practice regarding intravenous therapy found that training on IV fluid therapy was a major predictor factor knowledge of nurses^16^.

Our study identified that only 25.9% of nurses correctly checked respiration, and auscultated lung sounds periodically. Similarly, a study conducted in Egypt to assess the nursing efficiency of IV fluid therapy in pediatrics revealed that few nurses (18%) correctly checked respiration, and auscultated lungs periodically^22^.

The current study showed that training, level of education of nurses, and work experience had no significant association with the practice level of nurses regarding IV fluid therapy. Opposite to this result study conducted in Kenya to assess nurses' Competence in intravenous fluid therapy in under-fives with dehydration reported that training affected their competence in IV fluid therapy in 75%, and work experience in 56.5%^12^.

The current study showed that 56.3% of respondents had a satisfactory practice of IV fluid therapy in children. This result was consistent with one study on nurses' KAP concerning intravenous fluid therapy in children in which 60.4% of nurses had a satisfactory practice. Contrary to our report one study showed that only 7% of nurses had good practice in IV fluid therapy in children. This difference could be attributed to the researcher's inclusion of nurses working in a private hospital in the study^23^.

The present study also showed that the majority (89.3%) of studied nurses correctly checked physician orders, and 84.8% of them documented any additives to IV fluid. This result was aligned with the study employed in Trivandrum to assess the knowledge and practices of staff nurses regarding fluid in cardiac ICU and surgical wards in which about 91.8% of nurses checked physician orders, and 76.7% documented any additives added to IV fluid^24^.

In the present study, there was no statistically significant association between gender and the practice level of a nurse with P < 0.05. Consistently studies conducted in Iraq showed that there was no association between the practices of nurses and their gender concerning fluid therapy in children. This study also reported that there was a significant association between nurses' age, years of experience, and level of education at a P value of < 0.05. However, our study found that there was no significant relationship between age, years of experience, and practice level of respondents regarding IV fluid therapy in children^23^. The difference may have been due to lower in-service training in our study setup.

The present study revealed that there was no statistically significant association found between nurses' knowledge scores and practice scores. The result contradicts the study employed in Kenya on the assessment of nurses’ competence on intravenous fluid therapy in under-fives with dehydration that showed a significant association between knowledge and practice of IV fluid therapy with a P < 0.05^12^. This could be attributed to educational and/or training strength in the coordination of practice and knowledge in the latter study.

## Limitations of the study

The study has small sample size for further generalization. The study did not include private hospitals. It was limited to only nurses working in the pediatric emergency department.

## Conclusion and recommendations

More than half of nurses had inadequate knowledge regarding intravenous fluid therapy in children. Nurses' practice was relatively satisfactory concerning fluid therapy in children. A significant association was observed between nurses' intravenous fluid therapy knowledge and in-service training on intravenous fluids, educational qualification, and training institute. There should be targeted educational intervention strategies on IV fluid therapy through continuing education sessions, and on-site and off-site training with a specific focus on IV fluid therapy in children, their indications, and monitoring for associated complications. There is a need for further studies incorporating nurses working in private hospitals and other wards of the hospitals. Other study should also be performed to investigate other associated factors that cause nurses’ inadequacy of knowledge and unsatisfactory practice such as work overload, job satisfaction and promotion.

### Clinical significance and relevance

Adequate knowledge and satisfactory practice regarding intravenous fluid therapy among nurses are crucial in ensuring quality healthcare, reducing intravenous-related morbidity and mortality, and improving patient outcomes.

## Data Availability

The datasets used and/or analyzed during the current study are available from the corresponding author upon reasonable request.

## Abbreviations

AOR: Adjusted odd ratio
CI: Confidence interval
COR: Crude odd ratio
ED: Emergency Department
